# Supplementary material for: Efficiency of a music-based intervention as an adjunct to the first noninvasive ventilation session in acute exacerbation of COPD: A randomized single-blind controlled trial protocol
Source: PLoS One. 2025 Jul 23;20(7):e0328558. doi: 10.1371/journal.pone.0328558 (PMC12286333; doi:10.1371/journal.pone.0328558)
Supplement: S2 File — (PDF) [file pone.0328558.s002.pdf]

UNIVERSITE DE SOUSSE
FACULTE DE MEDECINE
"IBN EL JAZZAR" DE SOUSSE
COMITÉ D'ETHIQUE MEDICALE

جامعة سوسة
كلية الطب "ابن الجزار"
سوسة
لجنة الاخلاقيات الطبية

*** *** *** *** *** *** *** **** *** ***

Sousse, le .... 14 DEC. 2024

Réf : CEFMSo_0037_2024

OPINION OF THE COMMITTEE OF MEDICAL ETHICS

Object: Ethical opinion on the study entiltled: « Effectiveness of music therapy on the efficacy of noninvasive ventilation in acute exacerbation of chronic obstructive pulmonary disease: a randomized controlled clinical trial protocol ».

The Medical Ethics Committee of the Faculty of Medicine "IBN EL JAZZAR" of Sousse, was referred for an ethical opinion relating to the above-mentioned project.

The committee adopted the following decision: « **FAVORABLE OPINION**»

Chairman of the committee

Dr. Maher JEDIDI
Professeur en Médecine Légale

University of Sousse
Faculty of Medicine
«Ibn El Jazzar» of Sousse
COMMITTEE OF MEDICAL ETHICS

Adresse: Rue Mohamed Karoui Sousse - 4002 Tunisie
Site web: https://www.medecinesousse.com
Téléphone: +216 73 222 108
Email: comité.ethiquefmso@famso.u-sousse.tn

Human Participants Research Checklist

Complete the following if your study involved human participants or human participants' data. These questions should be addressed for prospective and retrospective studies.

1. Did you obtain ethics approval for this study?
   - If yes, please upload (file type "Other") the original approval document you received from your ethics committee. <u>If the original document is in another language, please also provide an English translation.</u>

     _x__ Uploaded ___ N/A

     ⇒ **Yes, the study was ethically approved, and the approval letter is uploaded in the recommended file type (other)**
   - If you did not obtain ethical approval, please explain why this was not required below.

2. If you prospectively recruited human participants for the study – for example, you conducted a clinical trial, distributed questionnaires, or obtained tissues, data or samples for the purposes of this study, please report in the Methods:

   i. the day, month and year of the **start and end** of the recruitment period for this study.

   ii. whether participants provided informed consent, and if so, what type was obtained (for instance, written or verbal, and if verbal, how it was documented and witnessed). If your study included minors, state whether you obtained consent from parents or guardians. If the need for consent was waived by the ethics committee, please include this information.

   ___ Completed **__x_ N/A**

   This study is a protocol. Patients have not yet been enrolled.

3. If you are reporting a retrospective study of medical records or archived samples, please report in the Methods section:

   i. the day, month and year when the data were accessed for research purposes

   ii. whether authors had access to information that could identify individual participants during or after data collection

   ___ Completed **__x_ N/A**

UNIVERSITY OF SOUSSE
IBN EL MEDICAL SCHOOL
JAZZAR" SOUSSE

MEDICAL ETHICS COMMITTEE

جامعة سوسة
كلية الطب "ابن الجزار"
سوسة

لجنة الاخلاقيات الطبية

REQUEST FOR OPINION FROM THE ETHICS COMMITTEE

(Research project)

**Secretariat only:**
**Date of submission:** **14/10/** 2024 Reference: **CEFMSo_ 0037-2024**

**Titre du projet:** Effectiveness of music therapy on the efficacy of noninvasive ventilation in acute exacerbation of chronic obstructive pulmonary disease: a randomized controlled clinical trial protocol		
**Type of study**	Retrospective study: ☐ Prospective study: ☐<br>Observation study: ☐ Intervention study: ■<br>Autres (Préciser) :A randomized controlled clinical trial protocol	
**Scope of the study**	National research programme ☐ International ☐<br>Thesis of medicine ☐ Master/CEC of medicine ☐ Theses of science ☐<br>Other (Please specify):...... **research article**.............................	
**Principal Investigator**: project leader	**Researcher:**	
Identity/signature stamp/tel/mail<br>Name and surname: **Imen Ben Saida**<br>E. Mail : **imen.bensaida@yahoo.com**	**Name and surname: Imen Ben Saida**<br>**Status: Associate Professor**<br>**Adress : intensive care unit, Farhat Hached hospital, Sousse, Tunisia**<br>**Phone number:** +21622897096<br>**E. Mail : imen.bensaida@yahoo.com**	
**Submission to a CE**	☐ Yes (attach a copy of the notice) Non ☐	

**Context of research**
**Problem** (individual and/or community impact)
Chronic obstructive pulmonary disease (COPD) is a major global health concern, ranking as the third leading cause of death worldwide [1]. Non-invasive ventilation (NIV) is a cornerstone therapeutic strategy for managing patients with severe exacerbations, particularly those with hypercapnic encephalopathy [3]. However, the efficacy of this

technique is dependent on several factors. In fact, patient anxiety and discomfort, despite appropriate pressure settings and equipment, can lead to premature mask removal and excessive air leaks [4]. In recent years, there has been growing interest in applying music therapy in medical contexts, particularly in intensive care units. Recent studies have explored the potential benefits of music therapy in patients with acute respiratory failure requiring invasive mechanical ventilation [6–8]. Some studies have demonstrated positive effects of music therapy on anxiety, pain, analgesic use, delirium, and overall patient satisfaction [6,7,9,10]. Music therapy can also reduce agitation in confused patients, improve mood, and facilitate communication [6]. Within the context of NIV, music therapy may serve as a valuable non-pharmacological intervention. By enhancing patient acceptance and tolerance of the technique, music therapy can potentially improve the efficiency of NIV sessions [11].There is limited literature on the impact of music therapy on NIV experience. To the best of our knowledge, no studies have yet examined the intra-session effects of music therapy on critically ill COPD patients undergoing NIV for acute exacerbation. By addressing the psychological and emotional challenges associated with NIV, music therapy could potentially improve patient acceptance, adherence to this technique and then clinical outcomes.

Objective(s) of the work:

to assess the impact of music therapy on the efficacy of NIV in patients with Acute Exacerbation of Chronic Obstructive Pulmonary Disease (AECOPD). We hypothesize that intra-session music therapy can improve NIV efficacy and tolerance in AECOPD compared to conventional care.

Methodology (briefly)

1-Study type:

Mono-center, prospective, randomized, single-blinded, parallel-group trial

Location of study: Intensive Care Unit (ICU) of Farhat Hached Hospital in Sousse, Tunisia

Period: From February 2025 to August 2025

2-Population under study:

Target population:

- ***Inclusion criteria:*** Consecutive adult patients (≥18 years) with COPD admitted to the ICU for an AECOPD

will be included. Eligibility requires meeting the GOLD criteria for COPD, having a pH <7.35, and being assessed as suitable for NIV by the attending physician.

- ***Exclusion Criteria:***
  - Patients unable to tolerate NIV or for whom NIV is contraindicated
  - Patients with severe hearing impairment or deafness
  - Patients for whom life-sustaining therapies have been withdrawn
  - Patients who have received antidepressant or antipsychotic medications in the previous two months
  - Aversion of music as assessed by personal interview

Sampling procedure

Randomization: All inclusion and exclusion criteria will be thoroughly reviewed. Eligible participants will then be randomly allocated in a 1:1 ratio to either the intervention group (music) or the control group using a computer-generated random sequence to ensure allocation concealment and minimize selection bias.

Blinding of the participants is not possible due to the nature of the intervention; however, primary outcome assessors and data analysts will be blinded to group allocation to reduce assessment and analysis bias according to the prospective randomized open blinded endpoint (PROBE) method.

Sample size:

the final sample size is set at 38 patients per group in either a 'musical intervention' group or a 'control' group, Recruitment is planned over a 6-month period, with a target enrolment of 76 participants.

3-Data collection:

Measurement instrument/data collection sheet

Day 1: baseline assessment (t_0)

Before randomization, demographic and baseline measurements will be collected for each patient, including sociodemographic data (such as age and sex), the COPD Assessment Test (CAT), and modified Medical Research Council (mMRC) scores, along with baseline characteristics including the Charlson Comorbidity Index (CCI) and Simplified Acute Physiology Score II (SAPS II).

After the baseline assessment, participants will be randomised into one of the two groups.

During NIV session

Primary and secondary outcomes will be collected by the principal investigator, who will conduct all assessments while remaining blind to the participants' group allocations, data collection, and analysis.

Primary Endpoint

The primary outcome of the study will be the efficacy of NIV measured by the reduction in arterial Pressure of Carbon dioxide (PCO_2). This will be calculated as the difference between baseline PCO_2 and the PCO_2 levels recorded at specific time points during the NIV session (h2, h4): ***ΔPCO_2=Baseline PCO_2 (t_0) –Endpoint PCO_2 (t_1, t_2).***

Secondary Endpoints

- Unplanned interventions: The number of unplanned interventions required by caregivers during the NIV session will be documented.
- Changes in respiratory and cardiovascular parameters: These parameters will be assessed before the NIV session, immediately after proper setup (5 minutes), and then at hourly intervals (h1, h2, h3, h4):
  - Respiratory rate (RR)
  - Peripheral oxygen saturation (SpO2)
  - Tidal volume (Vt)
  - Leak rate
  - Heart rate (HR)
  - Systolic blood pressure (SBP)
  - Diastolic blood pressure (DBP)
- Respiratory comfort: assessed using a digital visual scale, changes in respiratory comfort will be measured (h0, h2, h4).
- Neurological assessment (h0, h2, h4):
  - Encephalopathy score (Kelly-Matthay scale)
  - Glasgow Coma Scale

- Richmond Agitation-Sedation Scale (RASS)

- Patient-reported outcomes (h0, h2, h4):
  - Borg Dyspnea Scale (BDS)
  - Verbal Numeric Rating Scale (VNS) for pain assessment
- Psychological assessment (h0, h4):
  - Rapid Clinical Test For delirium (4 AT)
  - State Trait Anxiety Inventory (STAI)
  - Spielberger's trait anxiety inventory
  - Perceived stress scale (PSS)

Statistical analysis and expected results

The statistical analysis will be conducted using SPSS version 20. All continuous variables will be expressed as mean ± SD or median with interquartile range (IQR) (25th-75th percentile), depending on the normality of distribution assessed by the Shapiro-Wilk test. Categorical variables will be summarized as frequencies and percentages.

For the primary endpoint, the mean reduction in PCO_2 between the intervention (music therapy) and control groups (ΔPCO_2= baseline PCO_2 – endpoint PCO_2) will be compared using a student t-test or Mann-Whitney U test, as appropriate. Repeated measures analysis of variance (ANOVA) or a mixed-effects model approach will be used to analyze within-group changes across time points (h1, h2, h3, h4) ensuring adjustments for potential correlations within repeated measures.

For secondary outcomes:

- Respiratory and cardiovascular parameters: Differences between groups over time will be analysed using repeated measures ANOVA or mixed-effects models.
- Respiratory comfort and patient-reported outcomes (e.g., BDS, VNS): group comparisons will be performed using t-tests or Mann-Whitney U tests for continuous variables, with effect sizes and 95% confidence intervals reported.
- Neurological and psychological assessments: changes from baseline (h0) to endpoint (h4) will be analysed

using paired t-tests or Wilcoxon signed-rank tests within groups, and independent t-tests or Mann-Whitney U tests between groups.

- For categorical secondary outcomes (e.g., unscheduled interventions): comparisons between groups will be made using a chi-square test or Fisher's exact test where appropriate.

Missing data will be handled using multiple imputation techniques if the proportion of missing data is significant, ensuring unbiased estimates. Sensitivity analyses will be performed to assess the robustness of the results, particularly for the primary outcome.

All statistical analyses will be conducted on an intention-to-treat basis at a bilateral 5% alpha risk. Results will be interpreted with both statistical significance ($p < 0.05$) and clinical relevance considered. Analyses will be performed by an independent investigator blinded to treatment allocation, ensuring unbiased interpretation of the data.

Sources of project funding

No ■

Research laboratory: ....

Other: ....

Conflict of interest

Non ■

Yes☐ **if yes, declare** ....

....
