# Supplementary material for: Efficiency of a music-based intervention as an adjunct to the first noninvasive ventilation session in acute exacerbation of COPD: A randomized single-blind controlled trial protocol
Source: PLoS One. 2025 Jul 23;20(7):e0328558. doi: 10.1371/journal.pone.0328558 (PMC12286333; doi:10.1371/journal.pone.0328558)
Supplement: Attachment — Submitted filename: Responses to reviewers Final+++.docx [file pone.0328558.s003.docx]

Dear Editor-in-Chief of “**Plos One**”,

I would like to extend my gratitude to the editorial board and reviewers for their time and careful consideration of our manuscript entitled "**Effectiveness of music therapy on the efficacy of noninvasive ventilation in acute exacerbation of chronic obstructive pulmonary disease: a randomized controlled clinical trial protocol** ".

All necessary changes were brought to this manuscript, taking into account the comments of the reviewers.

We hope that this manuscript will be eligible for publication in your reputed journal.

Sincerely yours**,**

| Reviewer comments for:**Manuscript ID PONE-D-25-06120.** | | |
| --- | --- | --- |
| **Comment** | **Author Response** | **Text Insertion (if applicable)/ page/ line number of change** |
| **REFEREE 1** | | |
| .The protocol would benefit greatly from a conceptual framework to guide the music selection, the frequency and length of intervention, the proposed mechanisms of action on pCO2. | .Thank you for your valuable feedback regarding the need for a more explicit conceptual framework. We have addressed this in the revised manuscript by developing a detailed framework that outlines the theoretical basis for music's potential impact on pCO2. This framework integrates existing literature on music-based interventions, respiratory mechanics, the equation of motion of the respiratory system, respiratory physiology, and the autonomic nervous system to support our hypotheses. We believe these revisions provide a more robust conceptual framework for our study. | **.We added the following paragraph to the introduction section**  **Line 92-110**  *“In managing severe AECOPD with NIV, the primary aim is to offset the increased resistive load imposed by the exacerbation. Physiologically, the total pressure required for breathing (Ptot) is the sum of the pressure generated by respiratory muscles (Pmusc) and the pressure applied to the airways (Paw). The goal of NIV is to balance the recruitment of maximal Pmusc without inducing fatigue, achieved by providing adequate pressure support (PS) while minimizing leaks. We hypothesize that this equilibrium and the overall efficiency of NIV can be enhanced by incorporating music. Music, particularly with a tempo between 60 and 80 bpm, has been shown to significantly affect respiration by promoting relaxation and stabilizing vital signs [19,20]. It stimulates the limbic system, leading to endorphin release and a sense of well-being [20,21]. Furthermore, some research suggests music can influence serotonin production, which may enhance respiratory patterns, leading to improved breathing rates and depth [21]. A systematic review and metanalysis by Huang et al. [22]suggested that music-based intervention can enhance potential therapeutic effects in COPD patients.*  *By addressing the psychological and emotional barriers to effective NIV (anxiety, discomfort, poor tolerance), intra-session music-based intervention could potentially improve patient acceptance and adherence to the technique. This, alongside the direct physiological effects of music on the respiratory system and vital signs, can in turn contribute to a more relaxed and efficient respiratory pattern, indirectly enhancing ventilation efficiency and arterial partial pressure of carbon dioxide (PaCO₂) clearance.”* |
| .As written, it is not clear why a 2-hour testing period was selected nor is there sufficient detail about the music selected for intervention (who selected, characteristics, genre, etc). | .Thank you for raising this important point regarding the selection of a 2-hour testing period for the music-based intervention.  Initially, we considered providing music for the entire duration of the first NIV session (4 hours), aligning with the 2–4-hour timeframe within which a positive physiological response to NIV is typically observed [1,2]. However, we subsequently conducted a preliminary feasibility study on a small cohort of patients undergoing NIV. The results of this pilot work indicated that a 2-hour music intervention was both well-tolerated and effective in maintaining patient comfort and engagement throughout the session, without causing an undue burden or fatigue.  We acknowledge that the optimal duration of music intervention is not yet definitively established in the literature, with previous studies ranging from 10 minutes to 2.5 hours **[3]**.  A previous trial by Messika et al.**[4]**, exploring the effect of a musical intervention on respiratory comfort during NIV in ICU patients with acute respiratory failure, failed to show a statistically significant reduction in respiratory discomfort. The authors suggested that a 30-minute intervention might have been too short, representing a potentially "under-dosed" music intervention.  Furthermore, this duration allowed for a meaningful application of the 'U' sequence, facilitating both deep relaxation and gentle re-energization phases, while also being practical within the clinical workflow and the patients' tolerance levels during NIV.  **Therefore,** the 2-hour duration was empirically determined based on our initial feasibility assessment to balance potential therapeutic benefits with patient comfort and adherence within the specific context of NIV. Furthermore, a literature search and the previous trial by Messika et al. also informed this decision.  .The music playlist in the present study was carefully curated by WJ, a highly qualified music therapist and Founder/Coordinator of the Music Therapy Specialized Master at the Faculty of Medicine of Sousse, holding a PhD in Cultural Sciences specializing in Psychology of Music, Musicology, and Music Therapy. This curation was not arbitrary but followed consultation with the steering committee (IB, MB), experienced intensivists specializing in respiratory resuscitation who are accustomed to managing severe exacerbations of COPD. MB is the founder of the Non-Invasive Ventilation Specialized Master at the Faculty of Medicine of Sousse, and IB is the Coordinator.  Regarding music selection, we have significantly expanded this section to clarify our process. The characteristics of the selected music are now more comprehensively described within the manuscript, and the complete playlist has been added as a reference in Zenodo. | **.The paragraph was modified line 230-255**  *“Aligning with the 2–4-hour timeframe within which a positive physiological response to NIV is typically observed [4,9], the duration of the music-based intervention is set at two hours. This decision was informed by the results of a preliminary feasibility study conducted on a small cohort of patients. This pilot study demonstrated that a two-hour duration was both well tolerated and effective in maintaining patient comfort and engagement, without undue burden. The optimal duration of music intervention is not yet definitively established in the literature, with previous studies ranging from 10 minutes to 2.5 hours [26]. Furthermore, a previous trial by Messika et al.[16], exploring the effect of a musical intervention on respiratory comfort during NIV in ICU patients with acute respiratory failure, failed to show a statistically significant reduction in respiratory discomfort. The authors suggested that a 30-minute intervention might have been too short, representing a potentially 'under-dosed' music intervention. Patients will retain control over the intervention, with the option to pause or stop the music at any time by signalling the researcher.*  *Given the practical limitations of conducting standard psycho-musical assessments for critically ill COPD patients, a qualified music therapist (WJ in the authors’ list) created a customized music playlist for this study in consultation with experienced intensivists specializing in respiratory resuscitation (IB and, MB in the authors’ list) [27].*  *This playlist utilizes the 'U' sequence, a standardized receptive music-based intervention technique developed at the University Hospital of Montpellier and designed to induce relaxation through a gradual progression of music. This sequence begins with calming melodies and gradually increases in tempo and intensity, creating a "U-shaped emotional curve" where a downward phase reduces musical elements to promote deep relaxation, followed by an upward re-energizing phase [28,29]. This approach aims to reduce anxiety and promote relaxation in COPD patients experiencing hypercapnic acute respiratory failure undergoing NIV and has also been shown to enhance the caregiver-patient “listening” relationship [30], which is crucial for patient acceptance and adherence to NIV. “*  **.The following paragraph was added Line 243-246**  **“***Given the practical limitations of conducting standard psycho-musical assessments for critically ill COPD patients, a qualified music therapist (WJ in the authors’ list) created a customized music playlist for this study in consultation with experienced intensivists specializing in respiratory resuscitation (IB and, MB in the authors’ list) [27]”*  **We added the following paragraph line 256-269**  “*To maximize acceptability within a diverse Tunisian cohort (varying in education, musical background, and regional culture), the playlist features familiar and generally well-tolerated Tunisian and Oriental instrumental pieces (oud, qanun, nay, violin), including modal improvisations and a cappella segments suitable for COPD patients generally aged 45 and above. This culturally resonant repertoire incorporates specific musical attributes designed to enhance physiological and emotional well-being, including a slow tempo (60–80 BPM) to align with resting heart rate and promote parasympathetic activation, steady, cyclical rhythms (around 6 breaths/min) to stabilize respiration and improve heart-rate variability, simple consonant maqam improvisations (Nahawand, Ajam, Hajaz, Rast, et Bayati) to minimize cognitive load and facilitate relaxation, and soft instrumental timbres with occasional a cappella to reduce sympathetic activity and cortisol levels. Furthermore, a gradual decrease then increase in dynamics and orchestration (downward then upward U-phase) intends to guide an emotional arc from relaxation to gentle re-activation, potentially supporting NIV acceptance and adherence.”* |
| .It is not appropriate to use the term music therapy if the intervention was not delivered by a board-certified music therapist who interacted with the participants. | .Thank you for your valuable feedback regarding the use of the term 'music therapy.'  The music playlist in the present study was carefully curated by WJ, a highly qualified music therapist. This curation was not arbitrary but followed consultation with intensivists specializing in respiratory resuscitation who are accustomed to managing severe exacerbations of COPD.  We have replaced the term 'music therapy' throughout the manuscript with 'music-based intervention.' We believe this revised terminology appropriately conveys the standardized nature of the musical intervention delivered in this study. | ."music therapy" has been replaced with "*music-based intervention*" throughout the manuscript and the title. |
| .There is no coherent conceptual basis for why music should reduce pCO2 levels among patients with NIV. | .As stated in our response to the first remark, a detailed framework outlining the theoretical basis for music's potential impact on pCO2 has been added to the introduction section.  The objective of NIV in AECOPD patients with hypercapnic encephalopathy is to reduce pCO2 and normalize pH, along with improving the patients’ work of breathing.  Our conceptual framework posits that music-based intervention can influence factors that significantly impact the efficiency of NIV and, consequently, pCO2 levels.  As stated in **Line 61**, patient anxiety and discomfort are major barriers to successful NIV, often leading to premature mask removal and air leaks, thereby hindering effective ventilation and CO2 clearance. By mitigating these barriers, we hypothesize that patients will be more likely to tolerate the NIV interface for the prescribed duration, leading to more effective ventilation and subsequent CO2 removal. Guidelines from learned societies in intensive care **[2]** encourage the use of non-pharmacological interventions instead of sedation to optimize the efficiency of NIV. | .We have added the following paragraphs  ***Line 65-68**  “*The clinical challenges of NIV intolerance sometimes lead to light sedation for comfort and interface management [8]. While potentially helpful, sedation carries risks like respiratory depression, hypotension, and delirium [8]. Therefore, non-pharmacological interventions may be of great interest [9].”*  ***Line 92-110**  *“In managing severe AECOPD with NIV, the primary aim is to offset the increased resistive load imposed by the exacerbation. Physiologically, the total pressure required for breathing (Ptot) is the sum of the pressure generated by respiratory muscles (Pmusc) and the pressure applied to the airways (Paw). The goal of NIV is to balance the recruitment of maximal Pmusc without inducing fatigue, achieved by providing adequate pressure support (PS) while minimizing leaks. We hypothesize that this equilibrium and the overall efficiency of NIV can be enhanced by incorporating music. Music, particularly with a tempo between 60 and 80 bpm, has been shown to significantly affect respiration by promoting relaxation and stabilizing vital signs [19,20]. It stimulates the limbic system, leading to endorphin release and a sense of well-being [20,21]. Furthermore, some research suggests music can influence serotonin production, which may enhance respiratory patterns, leading to improved breathing rates and depth [21]. A systematic review and metanalysis by Huang et al. [22]suggested that music-based intervention can enhance potential therapeutic effects in COPD patients.*  *By addressing the psychological and emotional barriers to effective NIV (anxiety, discomfort, poor tolerance), intra-session music-based intervention could potentially improve patient acceptance and adherence to the technique. This, alongside the direct physiological effects of music on the respiratory system and vital signs, can in turn contribute to a more relaxed and efficient respiratory pattern, indirectly enhancing ventilation efficiency and arterial partial pressure of carbon dioxide (PaCO₂) clearance.”* |
| .Justification for the application of headphones only needs to be strengthened. | .The application of headphones in the control group (receiving NIV only) is a deliberate methodological choice aimed at standardizing the experience across both study groups and minimizing potential confounding factors. Furthermore, this choice was made to enhance the objectivity of outcome assessment and to ensure that both caregivers and data collectors remain blinded to group allocation throughout the study, in accordance with the principles of the PROBE design. | ***.The following paragraph was added line 223-226***  *“The application of headphones in the control group is a deliberate methodological choice aimed at standardizing the experience across both study groups and minimizing potential confounding factors by controlling for the presence of headphones and the procedural aspects.”* |
| .Efficiency and efficacy of music intervention is used interchangeably; only include one and fully define what is meant by efficiency. | .Thank you for pointing out the interchangeable use of "efficiency" and "efficacy." We have addressed this by consistently using "efficiency" throughout the revised manuscript to refer to the ability of the music-based intervention to produce the desired effect on the primary outcome (reduction in arterial PCO₂ at H4 of NIV).  . As requested, we have also included this definition of "efficiency" within the context of NIV in AECOPD patients with hypercapnic encephalopathy in the revised manuscript to ensure precision and avoid confusion. | **.” *Efficacy”* was replaced by *“efficiency” through the manuscript and in the title***  .The following paragraph was added **Line 55-60**  *“Efficiency of NIV in acute exacerbation of COPD (AECOPD) patients with hypercapnic encephalopathy refers to how quickly and effectively NIV achieves its intended physiological effect of improving gas exchange. It is worth noting that an improvement in gas exchange is a strong predictor of a successful outcome with NIV; in patients who respond, this improvement is almost universally observed within the first 2–4 hours after NIV initiation [4,5].”* |
| An intervention fidelity monitoring plan would add rigor to this protocol including who is responsible for delivery of the assigned condition, is it one time only or is it repeated, and how data are collected in a reliable and valid manner on all the outcomes. | We agree that ensuring standardization and adherence to the intervention protocol is essential for the internal validity of the study. We have now included a detailed fidelity monitoring strategy in the revised manuscript to address this concern. These additions specify the personnel responsible for the delivery of the intervention, frequency and duration of sessions, documentation of session duration and interruptions, and the approach for handling sessions that are shorter than the intended 2-hour period, both in data collection and in the analysis phase. | ***The following paragraph was added line 311-324***  ***“Intervention Fidelity and Data Quality Assurance:***  *A standardized protocol governs the delivery of the music-based intervention, ensuring consistency across all sessions. NIV will be managed by ICU staff according to routine clinical care practices. A comprehensive fidelity monitoring plan will be implemented to ensure consistent and reliable delivery of both the intervention and control conditions. This plan addresses key fidelity domains, including adherence, delivery quality, and data accuracy. After each session, senior physicians will complete a fidelity log documenting session duration, deviations, interruptions, patient engagement, and environmental factors.*  *The music-based intervention will be administered only once per participant, during the first NIV session, ensuring uniform exposure across groups. Sessions lasting less than the intended duration of two hours will be recorded, with reasons documented in the fidelity log. Data quality will be ensured through double data entry and verification processes.”*  *This structured fidelity and quality assurance approach is designed to uphold the trial’s internal validity and support accurate interpretation of the intervention’s effects****.”*** |
| .Details are needed to ensure adherence to a 2-hour intervention period and how shorter sessions are managed with measuring outcomes and how managed in the analysis. | .Thank you for raising this important point. We agree that patient adherence is a critical aspect of the intervention. In response, we have added a paragraph in the Methods section detailing the strategies that will be implemented to ensure adherence to the 2-hour music therapy session. These include supervision by the trained healthcare professional delivering the intervention, optimization of patient comfort and environmental conditions, regular checks of headphone placement and sound delivery, as well as documentation of any interruptions and the actual duration of exposure.  Furthermore, our protocol specifies that only sessions lasting the full intended duration of two hours will be included in the primary analysis of outcomes. Sessions falling below this threshold will be recorded, and the reasons for shorter durations will be documented in fidelity logs. While these sessions will be excluded from the primary efficacy analysis, a sensitivity analysis is planned to explore their potential impact on the outcomes. This approach ensures that the primary analysis focuses on adequately delivered sessions, thereby preserving the internal validity of the study. Details regarding this handling of shorter sessions and the sensitivity analysis have been included in the Statistical Analysis section of the revised manuscript. | ***.The following paragraphs were added :***  **Line 270-279:**  ***“Strategies to improve adherence to interventions:***  *To ensure adherence to the intended two-hour music-based intervention, several measures will be implemented. The healthcare professional administering the intervention, part of the core team of senior physicians, will supervise each session. This team will be trained to ensure proper implementation and patient comfort throughout the session. Patients will be positioned semi-recumbently and provided with appropriate headphones. The environment will be kept as quiet as possible, and headphone placement and sound delivery will be regularly checked. Any interruptions due to urgent care or intolerance to NIV will be documented, and the actual duration of exposure to music-based intervention will be recorded for each patient to accurately assess adherence.”*  **Line 304-308:**  ***“****To ensure protocol fidelity and minimize inter-provider variability, this core team will deliver all intervention sessions according to shift schedules, using a timer to monitor session duration and document key session details. Outcome assessments will be conducted by four well-trained residents who are blinded to participants’ group assignments and data analysis****.”***  ***.Line 431-432:***  ***“****For the primary efficiency analysis, only sessions with the intended duration will be included.”*  *.* ***Line 469-477:***  *“Sensitivity analysis :*  *To evaluate whether session duration affects the primary outcome a sensitivity analysis will be performed including all recorded sessions, regardless of length. A separate multivariable linear regression model will be used, with ΔPaCO₂ as the dependent variable, group allocation as the main predictor, and session duration included as an additional covariate. The model will also adjust for the same baseline confounders (mMRC scale, familiarity with home NIV, obesity status, Kelly-Mathey score, and STAI-Trait score). This analysis will help determine whether the duration of the session significantly modifies the effect of the intervention on PaCO₂ changes.”* |
| .Additional details are needed to describe the setting from which patients are enrolled (type of unit, number of beds, staffing patterns, etc.). | .Thank you for highlighting the need for more details regarding the study setting. These details have been added to the revised manuscript. | **.The following paragraph was added Line 131-137**  “*Recruitment will take place in a 12-bed medical Intensive Care Unit (ICU) at Farhat Hached Hospital in Sousse, Tunisia. The medical staff comprises a department chief, three associate professors, three assistant professors, 12 ICU residents, and 4 interns supported by a paramedical team of 26 nurses and 2 physiotherapists. This unit primarily admits patients with AECOPD. A 4-bed unit is dedicated to NIV, with individualized rooms operating since the year 2000. NIV is a standard treatment modality within this unit and is routinely used for eligible patients according to established clinical guidelines.”* |
| .Details are needed on the how each of the variables of measured and with what specific instruments, including the validity and reliability. | .We have addressed the reviewer's request by providing detailed information on how each variable is measured, specifying the instruments used, and including details regarding their validity and reliability in the revised manuscript. These details are now included in the “Study endpoints” section of the manuscript to enhance transparency and reproducibility. | .We added the following paragraph **Line 331-384 :**  “…*PaCO₂ levels will be measured using the GEM Premier 3500 Blood Gas Analyzer, a device known for its fast, reliable, and efficient blood gas analysis. The GEM Premier 3500 has established validity and reliability for measuring blood gas parameters [31].*  ***Secondary Endpoints***  *•****The evolution of PaCO₂*** *during the first 4 hours of NIV. This will be assessed by measuring PaCO₂ at baseline, before NIV initiation (t0), at 2 hours (t2), and at 4 hours (t4).*  *•****Unplanned interventions:*** *The number of unplanned interventions required by caregivers during the NIV session will be documented through direct observation and review of nursing notes.*  *•****Changes in respiratory and cardiovascular parameters:*** *Respiratory rate (RR), peripheral oxygen saturation (SpO₂), heart rate (HR), systolic blood pressure (SBP), and diastolic blood pressure (DBP) will be continuously monitored and recorded using the Edan Elite V6 modular patient monitoring system. This system provides validated and reliable measurements of these physiological parameters. Tidal volume (Vt) and leak rate will be directly collected from the ventilator's monitoring system (Mindray SV 600 or Löwenstein Elisa 600). Measurements will be taken before NIV initiation (t0), and then at intervals (t2, t4).*  *•****Respiratory comfort:*** *will be assessed using a 100mm digital visual analog scale (VAS), where 0 represents "no discomfort" and 100 represents "maximum discomfort".Respiratory comfort will be measured at baseline t0, t2 and t4. The VAS is a commonly used and validated tool for subjective symptom assessment [32].*  ***•Neurological assessment*** *(t0, t2, t4) using the following validated scales:*  *-Encephalopathy score : The Kelly-Matthay scale, a 5-point scale specifically assessing the level of consciousness in hypercapnic encephalopathy, will be used. Its reliability and validity in this context have been described in the literature [33,34].*  *-Richmond Agitation-Sedation Scale (RASS) : : A validated 10-point scale used to assess the level of sedation or agitation in critically ill patients [35].*  ***•Patient-reported outcomes (t0, t2, t4):***  *-Dyspnea will be evaluated using the Borg Dyspnea Scale (BDS) :A validated 10-point scale ranging from 0 ("no breathlessness") to 10 ("maximal breathlessness "), reflecting the patient's subjective perception of shortness of breath. The BDS is a well-established and reliable measure of dyspnea [36].*  *-Pain intensity will be measured using Verbal Numeric Rating Scale (VNS): A 0-to-10 scale where 0 represents "no pain" and 10 represents "worst possible pain". Patients will verbally rate their current pain level. The VNS is a commonly used and accepted pain assessment tool [37].*  *•****Psychological assessment (t0, t4):***  *-Rapid Clinical Test For delirium (4 AT): A brief and validated screening tool for delirium [38].*  *-State Trait Anxiety Inventory (STAI): A widely used and validated self-report questionnaire measuring both state (current) and trait (general tendency) anxiety [39].Perceived stress scale (PSS): A widely used and validated 10-item questionnaire measuring the degree to which individuals perceive their lives as stressful [40].*  *Self-reported questionnaires (STAI and PSS) have been validated for use in Arabic-speak patients [39,40].*  *Data will be collected using a dedicated case report form. The completed forms will be sent to the data manager for anonymization and entry into a secure database. “* |
| .Unclear if the analysis will consider any covariates and how they will be addressed. | .Thank you for your insightful comment. We have clarified in the Statistical Analysis section that relevant covariates (e.g., baseline dyspnea severity assessed by the mMRC scale, familiarity with home NIV, obesity status, Kelly Mathey scale score, baseline anxiety trait (STAI trait score)) will be considered in the analysis. These variables will be included in multivariable models, where appropriate, to control for potential confounding. The manuscript has been updated to detail the process for incorporating covariates into the analysis. | ***We added the following paragraph line 433-442***  ***“Adjusted Primary Analysis:***  *To account for potential confounding variables, a multivariable linear regression model will be performed, with ΔPaCO₂ as the dependent variable and group allocation (intervention vs. control) as the main independent variable. The model will adjust for baseline factors known or suspected to influence PaCO₂ levels, including :*  *• dyspnea severity (assessed by the mMRC scale),*  *• prior use of home NIV,*  *• obesity status,*  *• Kelly Mathey scale score, and*  *• baseline anxiety trait (measured by the STAI trait score).”* |
| **REFEREE 2** | | |
| .I suggest some modifications  eligibility : why ph below 7.35 ,I suggest ph below 7.30 ( in this case NIV is mandatory ) | .We appreciate the reviewer's suggestion to lower the pH eligibility criterion to ≤ 7.30. However, our inclusion criterion of pH ≤ 7.35 is directly based on the strong recommendation with high certainty of evidence from the official European Respiratory Society/American Thoracic Society (ERS/ATS) clinical practice guidelines for NIV use in AECOPD patients with acute respiratory acidosis **[1]**. Adhering to these established guidelines ensures our study population aligns with current best practice for NIV initiation.  Therefore, we believe that maintaining the pH inclusion criterion of ≤ 7.35 aligns with the current best clinical practice guidelines and will allow us to investigate the effects of our intervention in AECOPD patients for whom NIV is strongly recommended. | *.*No action is needed |
| .kelly scale threshold should be included in the indication for NIV | . Thank you for raising this important point regarding the Kelly Mathey scale threshold in the indication for NIV. We appreciate the opportunity to clarify its role in our study.  While the Kelly Mathey scale was not a primary inclusion criterion for NIV initiation (which followed established clinical guidelines as mentioned in the manuscript), we did utilize it to characterize the severity of hypercapnic encephalopathy after randomization. This baseline assessment using the Kelly Mathey scale will be included as a covariate in our multivariable analyses to adjust for potential differences in the severity of encephalopathy between the intervention and control groups. This approach allows us to account for this important clinical factor when evaluating the effect of the music-based intervention on our outcomes.  We have added a clarification in the 'Statistical Analysis' section to explicitly state that the Kelly Mathey scale will be included as a covariate in the adjusted analyses. | **.The following paragraph was added Line 433-442**  ***“Adjusted Primary Analysis:***  *To account for potential confounding variables, a multivariable linear regression model will be performed, with ΔPaCO₂ as the dependent variable and group allocation (intervention vs. control) as the main independent variable. The model will adjust for baseline factors known or suspected to influence PaCO₂ levels, including :*  *• Dyspnea severity (assessed by the mMRC scale),*  *• Prior use of home NIV,*  *• Obesity status,*  *• Kelly Mathey scale score, and*  *• Baseline anxiety trait (measured by the STAI trait score).”* |
| .Why patients in treatment with chronic psycothropic drugs should be excluded? | . We thank the reviewer for raising this important point regarding the exclusion of patients on chronic psychotropic medications. This exclusion criterion was implemented to minimize potential variability within our study population and thereby improve the internal validity of our findings. These medications can exert diverse effects on sedation levels, agitation, cognitive function, and potentially respiratory drive, all of which are directly relevant to our primary outcome and several secondary outcomes, including neurological status (Encephalopathy score, RASS), respiratory comfort (Borg Dyspnea Scale), and psychological assessments (Rapid Clinical Test For delirium, State Trait Anxiety Inventory, perceived stress scale). By excluding this subgroup, we aimed to reduce the risk of these medications acting as confounding factors, allowing for a clearer and more direct assessment of the impact of music-based intervention on NIV efficiency and the other measured endpoints in a more consistent patient population. Furthermore, it can sometimes be difficult to distinguish between hypercapnic encephalopathy and psychiatric disorders, adding complexity to the assessment. After all, this situation (patients on chronic psychotropic medications) is relatively scarce among COPD patients requiring NIV. | .No action is needed. |
| .AECOPD standard therapy should be specified | .Thank you for your remark  .We have specified the standard therapy for AECOPD, which typically includes oxygen, bronchodilators, systemic corticosteroids, and antibiotics when indicated for bacterial infection. | ***.We added a paragraph entitled standard of care line 206-219***  *“****Standard of care:***  *All patients will receive NIV delivered through an oro-nasal mask (Fisher & Paykel) with a seal adapted to the patient's face, in a semi-recumbent position with the door closed and a calm environment, using either a Mindray SV 600 or a Lowenstein Elisa 600 ventilator, without an artificial nose or humidifier to limit instrumental dead space. PEEP will be set at 80% of auto-PEEP measured before the start of NIV (using a short expiratory pause of <0.3 seconds); if auto-PEEP cannot be measured, initial PEEP will be 3–5 cmH2O. Final PEEP and PS will be titrated based on individual needs and continuous ventilator waveform monitoring to optimize synchrony, tidal volume (target 6–8 mL/kg ideal body weight), and work of breathing. PEEP will be adjusted to counteract auto-PEEP, guided by waveform analysis [4,24]. Both groups will receive standard AECOPD therapy, which includes oxygen titrated to maintain a target oxygen saturation (SpO2) of 88–92%, bronchodilators, hydration, systemic corticosteroids, and antibiotics when indicated by signs of bacterial infection, in accordance with current clinical guidelines [25].”* |
| .Ventilator setting : why PEEP should be set at 3-5 cmH2O ( which rationale? )  Which type of ventilators will the authors use? PS and PEEP should be according to ventilator waves. | .We appreciate the reviewer's question regarding the rationale for setting PEEP at 3-5 cmH2O. This low-level PEEP is primarily intended to counteract intrinsic PEEP (auto-PEEP), a common finding in patients with AECOPD due to airflow limitation and incomplete exhalation. Applying external PEEP can help to counterbalance the PEEPi when initiating inspiration, thereby reducing the work of breathing and improving trigger synchrony. Furthermore, maintaining a slightly positive pressure can help keep airways open during expiration, reducing resistance and facilitating air exit from the lungs, potentially improving gas exchange and preventing alveolar collapse without risking overdistension.  This initial PEEP range of 3-5 cmH2O aligns with recommendations in the literature for starting NIV in AECOPD patients and aims to optimize patient comfort and ventilator synchrony while addressing the physiological challenges associated with this condition **[1,5]**. | **.The following paragraph was added line 206-219**  *“All patients will receive NIV delivered through an oro-nasal mask (Fisher & Paykel) with a seal adapted to the patient's face, in a semi-recumbent position with the door closed and a calm environment, using either a Mindray SV 600 or a Lowenstein Elisa 600 ventilator, without an artificial nose or humidifier to limit instrumental dead space. PEEP will be set at 80% of auto-PEEP measured before the start of NIV (using a short expiratory pause of <0.3 seconds); if auto-PEEP cannot be measured, initial PEEP will be 3–5 cmH2O. Final PEEP and PS will be titrated based on individual needs and continuous ventilator waveform monitoring to optimize synchrony, tidal volume (target 6–8 mL/kg ideal body weight), and work of breathing. PEEP will be adjusted to counteract auto-PEEP, guided by waveform analysis [4,24].”* |
| **REFEREE 3** | | |
| .The authors presented the study protocol of a prospective, randomized, single-blinded, parallel-group trial about the effectiveness of music therapy as add on therapy to noninvasive ventilation in acute exacerbation of chronic obstructive pulmonary disease. As primary endpoint variable serves the reduction in arterial Pressure of Carbon dioxide (PCO₂).  The protocol is partly sound from the methodological point of view. The trial is registered but not currently recruiting.  The protocol needs revision from the methodological point of view.  There are methodological issues with the description of the randomization process and the correct coherent statistical analysis taking sample size justification into account. Among others type I error probability, by various sources, is not controlled. As such the validity and reproducibility of the trial is not given. | .We thank the reviewer for the thoughtful and constructive comments regarding the methodological aspects of our study protocol.  We fully acknowledge the importance of ensuring a rigorous and transparent methodological framework to support the validity and reproducibility of our protocol. In response to your feedback, we have thoroughly revised the manuscript and addressed each of the concerns raised.  More specifically:   - The description of the **randomization process** has been clarified and expanded to provide greater transparency regarding allocation concealment and the sequence generation process. - The **primary outcome** has been clearly defined and refined to ensure alignment with the intervention and to improve clarity for readers and reviewers. - The **statistical analysis plan** has been revised to include a detailed explanation of the adjusted primary analysis using multivariable models, explicitly addressing how relevant covariates will be handled. - The issue of **type I error** has been addressed by clearly stating the significance threshold and explaining how multiplicity, if applicable, will be managed. - A **fidelity monitoring plan** and a **missing data management strategy** have been added to reinforce internal validity and ensure consistency in data collection and interpretation.   We hope these revisions enhance the methodological rigor and clarity of the study protocol and address your concerns comprehensively. All changes made in response to your comments are clearly indicated in the revised manuscript.  We remain grateful for your valuable input, which has significantly contributed to the improvement of our protocol.  If any of the revisions remain unsatisfactory or unclear, we are fully open to further clarification and modification to ensure the protocol meets the highest standards. | ***.*** *Modifications are detailed below.* |
| .L76: The statement can not be directly translated in a hypothesis. Major items of the PICO statement are missing. The classification of the hypothesis, e.g. ∂ equivalence is not given. | .Thank you for this important observation. We have revised the study objective to align clearly with the PICO framework and reformulated the hypothesis accordingly. We also clarified that this is a superiority trial. These changes improve the specificity and methodological transparency of the study. | **. Line 111-119:**  **“*Objective:*** *Based on this conceptual framework, we aim to assess whether incorporating an intra-session music-based intervention during the first NIV session for severe AECOPD management enhances its efficiency in reducing PaCO₂ as a primary outcome, compared to standard NIV therapy.*  ***Hypothesis:*** *We hypothesize that incorporating a 2-hour music-based intervention during the initial NIV session in adults hospitalized with AECOPD will result in a significantly greater reduction in PaCO₂ after 4 hours of NIV, compared to standard NIV care alone, supporting the superiority of the intervention over standard care.*” |
| .L94: The eligibility criteria lack of important exclusions, like age restrictions, pregnancy restriction, dependency restrictions, other trials, etc. This is even not included in the registration. | . We acknowledge that certain exclusions were not explicitly stated and will be added for clarity in the revised manuscript and trial registration.  Regarding the specific points raised:   - **Age Restrictions:** We would like to clarify that our inclusion criteria already specify "Consecutive adult patients (≥18 years) with COPD admitted to the ICU for an AECOPD will be included." This explicitly sets the lower age limit for participation.   Regarding an upper age limit, we did not impose one as the pathophysiology of AECOPD and the potential benefits of NIV and music-based intervention are not strictly limited by advanced age. Excluding older patients could limit the generalizability of our findings to the typical population suffering from severe AECOPD requiring ICU admission.   - **Pregnancy Restriction:** Our medical intensive care unit does not admit pregnant patients. Therefore, pregnancy is an implicit exclusion criterion. We will explicitly add "Pregnancy" to the exclusion criteria for absolute clarity. - **Other Trials:** To avoid potential confounding factors and ensure the integrity of our data, we will explicitly exclude patients currently participating in other interventional trials. This will be added to the exclusion criteria. - **Dependency restrictions** are not an exclusion criterion in the present study; however, the severity of the COPD patients' respiratory functional impairment, evaluated by the mMRC score, and prior use of home NIV will be considered in the analysis as covariates.   We appreciate the reviewer's diligence in identifying these omissions and will ensure these exclusions are updated in the trial registration to provide a more comprehensive understanding of our study population. | We added pregnancy and enrollment in other trials to exclusion criteria  **Line 146-154 :**  **“•*Exclusion Criteria:***  *-Patients unable to tolerate NIV or for whom NIV is contraindicated*  *-Patients with severe hearing impairment or deafness*  *-Patients for whom life-sustaining therapies have been withdrawn*  *-Patients who have received antidepressant or antipsychotic medications in the previous two months*  *-Aversion of music as assessed by personal interview*  *-Pregnancy*  *-Enrollment in other trials***”** |
| .L112: Process to preserve allocation concealment not clear. | . Thank you for your comment. We have clarified the procedure used to preserve allocation concealment in the revised manuscript. Group assignments will be securely stored ensuring that allocation remains concealed from the research team members involved in recruitment and assessment. Allocation concealment will be further maintained using sequentially numbered, sealed opaque envelopes containing each participant’s group assignment, along with a unique patient identifier code. | ***.* The following paragraph was added line 171-181**  *“****Concealment mechanism:*** *To ensure strict allocation concealment and prevent allocation bias, the randomization sequence will be securely stored and inaccessible to all study investigators and site staff involved in screening, enrolment, or intervention delivery. Group assignments will be concealed using a series of sequentially numbered, opaque, sealed envelopes prepared in advance. Each envelope will contain the participant's assigned group and a unique identifier code. Following the acquisition of written informed consent and confirmation of eligibility, a designated research nurse, who is not involved in participant recruitment or care, will open the corresponding sequentially numbered envelope to reveal the group allocation. This individual will be the sole person authorized to handle the sealed allocation envelopes. This procedure ensures allocation concealment until the point of randomization, maintaining methodological integrity and minimizing allocation bias. “* |
| .L113: Substitute selection by allocation | .We have replaced “selection” by “allocation” | ***.*Line 171**  Done |
| .L114: "Single randomization list" not clear. | .Thank you for pointing this out. We agree that the term “single randomization list” may be unclear. We have revised the wording to clarify that a single randomization sequence will be generated in advance for the entire study using a computer-based random number generator, with a 1:1 allocation ratio. This sequence will be used solely to prepare sealed, opaque envelopes for each participant. The sequence itself is not accessible to clinical staff or investigators, and allocation remains concealed until the point of randomization. Random block sizes are also used to further prevent predictability. | **.Line 165-170**  **“***This randomization will be conducted using a web-generated blocked randomization* ***sequence*** *via the Research Randomizer platform (www.randomizer.org), designed to maintain balanced group sizes throughout the trial.* ***The randomization sequence*** *will be generated in advance by a research assistant independent of participant recruitment, clinical care, outcome assessment, or data analysis*.” |
| .L114_116: Persons responsible and implement are not given. Specification necessary | . Thank you for your comment highlighting the need for clearer specification of the persons responsible for various aspects of the trial implementation. We have addressed this in the revised manuscript, and the roles are detailed below and within the 'Trial Implementation and Coordination' section:  **Trial Oversight and Coordination:** The overall conduct of the trial will be overseen by the site investigators (as mentioned in the manuscript for screening and enrolment).  **Randomization Sequence Generation**: A research assistant with no other study involvement will generate the randomization sequence.  **Screening and Enrolment:** Site investigators will be responsible for the daily screening and enrolment of eligible participants.  **Allocation Concealment**: A designated research nurse will perform the allocation by opening the sealed envelopes and will be the sole custodian of these envelopes.  **Intervention Delivery:** A core group of senior physicians will deliver the music-based intervention to the intervention group and facilitate the headphone use in the control group.  **Adherence Monitoring:** The senior physician delivering the intervention will be responsible for monitoring session length using a timer and documenting adherence in the study records.  **Outcome Assessment:** Well-trained residents blinded to group allocation will conduct the outcome assessments.  **NIV Management:** Routine clinical care and management of NIV will be the responsibility of the general ICU medical and paramedical staff, following established protocols.  **Statistical Analysis:** An independent investigator (DC in the authors’ list) blinded to treatment allocation will perform all statistical analyses using SPSS version 20.  We believe these modifications, included in the manuscript, clearly specify the individuals or groups responsible for each key stage of the trial, enhancing transparency and methodological rigor. | **. The following paragraph was added line 291-310**:  *“****Trial Implementation and Coordination***  *The rigorous implementation of this trial relies on strict protocol adherence clearly defined roles and standardized procedures to ensure methodological integrity and internal validity. Prior to enrolment, all ICU medical and paramedical staff will be trained on the study protocol and data collection methods during dedicated briefing sessions.*  *The randomization sequence will be generated by a research assistant not involved in recruitment, intervention delivery, or outcome assessment. Daily screening and enrolment of eligible participants will be carried out by site investigators. After obtaining written informed consent and confirmation of eligibility, a designated research nurse will perform the allocation by opening sealed, opaque envelopes containing the group allocation, serving as the sole custodian of the envelopes. Participants assigned to the intervention group will receive a single, two-hour music-based intervention during the first NIV session, administered by a core team of senior physicians. Control group participants will wear headphones without music for the same duration. To ensure protocol fidelity and minimize inter-provider variability, this core team will deliver all intervention sessions according to shift schedules, using a timer to monitor session duration and document key session details. Outcome assessments will be conducted by four well-trained residents who are blinded to participants’ group assignments and data analysis. To minimize bias, these residents will not be involved in recruitment or intervention delivery. All assessors will undergo specific training to standardize data collection procedures and reduce inter-rater variability. “*  **. Line 482-484**  *“The statistical analysis will be conducted using SPSS version 20. Analyses will be performed by an independent investigator (DC in the authors’ list) blinded to treatment allocation, ensuring unbiased interpretation of the data.”* |
| .L116: Substitute selection by allocation | .We have replaced “selection” by “allocation” | *Done*  ***Line 181*** |
| .L124: Statement about implementation of randomization list conflicts to concealment. | .Thank you for pointing this out. We have clarified the implementation of allocation concealment in the revised manuscript.  The randomization sequence is securely stored and is not accessible to study staff or investigators prior to participant enrollment. Allocation concealment is ensured by using sequentially numbered, sealed, opaque envelopes. After a participant has been enrolled and has provided consent, a designated research nurse, who is the sole custodian of these envelopes, will perform the allocation by opening the next envelope in the sequence. This research nurse is not involved in patient care, intervention delivery, or outcome assessment. | **.The paragraph was modified Line 296-303**  *“The randomization sequence will be generated by a research assistant with no involvement in recruitment, intervention delivery, or outcome assessment. Daily screening and enrolment of eligible participants will be carried out by site investigators. After obtaining written informed consent, a designated research nurse will perform the allocation by opening a sealed, opaque envelope containing the group allocation. This individual will be the sole custodian of the envelopes.*  *Participants randomized to the intervention group will receive a single, two-hour music-based intervention delivered by a core group of senior physicians, in parallel with NIV. Control participants will wear headphones without music for the same duration, also facilitated by the senior physicians.”* |
| .L128_131_ Process for implementation not clear. | . We appreciate the reviewer's comment regarding the clarity of the implementation process. To address this, we have provided a more detailed step-by-step description of the trial workflow in the section “trial implementation and coordination” in the revised protocol. | **. We added the following paragraph line 291-310**  *“****Trial Implementation and Coordination***  *The rigorous implementation of this trial relies on clearly defined roles and standardized procedures to ensure methodological integrity and internal validity. Prior to participant screening and enrolment, all ICU medical and paramedical staff will be trained on the study protocol and data collection methods during dedicated briefing sessions.*  *The randomization sequence will be generated by a research assistant with no involvement in recruitment, intervention delivery, or outcome assessment. Daily screening and enrolment of eligible participants will be carried out by site investigators. After obtaining written informed consent, a designated research assistant will perform the allocation by opening a sealed, opaque envelope containing the group allocation. This individual will be the sole custodian of the envelopes.*  *Participants randomized to the intervention group will receive a single, two-hour music-based intervention delivered by a senior physician, in parallel with NIV. Control participants will wear headphones without music for the same duration. A timer will be used to monitor session length and ensure adherence. To minimize inter-provider variability in the intervention delivery and maintain protocol fidelity, the same core group of senior physicians will be responsible for administering all intervention sessions throughout the trial, based on shift schedules. Well-trained residents will be the outcomes assessors.*  *The music-based intervention follows a standardized protocol to promote consistency across all sessions. NIV will be managed by ICU staff according to routine clinical care.”* |
| .L131: Reference for PROBE | .We have now included the full reference for the PROBE design in the manuscript (reference number 23). | ***.*Line 189**  ***Done.*** |
| L181: Primary endpoint variable unclear. Intervention is not an element of the definition. | We acknowledge that the initial phrasing may have lacked the necessary clarity and could have inadvertently implied the intervention as part of the endpoint definition.  To address this, we have revised the definition of the primary endpoint to focus solely on the physiological variable of interest, which is the change in PaCO₂. The revised statement will clearly define the primary endpoint as the absolute change in PaCO₂ from baseline to 4 hours of NIV, calculated as the difference between the PaCO₂ value measured at the start of the NIV session and the PaCO₂ value recorded at 4 hours.  The hypothesis, stated separately, will then articulate our expectation that the intra-session music-based intervention will lead to a significantly greater reduction in this primary endpoint (ΔPaCO₂) compared to standard NIV alone.  This separation will ensure that the intervention is recognized as the independent variable hypothesized to influence the primary outcome, rather than being part of its definition. | **The following paragraph was reformulated**  **Line 326-334**  ***“Primary Endpoint***  *The primary endpoint is the change in PaCO₂ from baseline to 4 hours after the initiation of NIV. ΔPaCO₂, calculated as the difference between PaCO₂ value measured at the start of the NIV session (t0) and PaCO₂ value recorded at 4 hours (t4): ΔPaCO₂ = PaCO₂ (t0) - PaCO₂ (t4). We hypothesize that the intra-session music-based intervention adjunct to NIV will lead to a significantly greater ΔPaCO₂ compared to standard NIV alone. PaCO₂ levels will be measured using the GEM Premier 3500 Blood Gas Analyzer, a device known for its fast, reliable, and efficient blood gas analysis. The GEM Premier 3500 has established validity and reliability for measuring blood gas parameters [31].”* |
| .If more than one timepoint is used methods to maintain type one error rate need to be specified. | .Thank you for this important comment. We have clarified how we will control the type I error rate in the revised manuscript.  To address this, we will use a linear mixed-effects model, which is a robust statistical method suitable for analyzing longitudinal data, especially when there are potentially unbalanced data with missing entries, and when measurements are repeated within individuals.  The change in parameters measured at several timepoint (at h0, h2, and h4) will be analyzed using this model, which accounts for the repeated measures within each participant and tests the group × time interaction. This approach directly assesses whether the evolution of these parameters change between groups across time points while controlling for within-subject correlation and repeated measures. The linear mixed effects model inherently controls the type I error rate for multiple timepoint comparisons by modeling the covariance structure of repeated measures and evaluating fixed effects globally. This approach avoids the need for separate pairwise comparisons and ensures a more accurate control of the error rate.  However, if post hoc comparisons (such as pairwise timepoint contrasts) are performed as part of exploratory analyses, a correction for multiple testing (e.g., the Holm-Bonferroni method) will be applied to maintain the overall type I error rate. | *.***L 444-463:**   - ***Change of PaCO₂ over time****: To evaluate the temporal evolution of PaCO₂ (at H0, H2, and H4), a*   *a linear mixed-effects model (also referred to as multilevel models) will be employed. This statistical approach is particularly suited to repeated measures data, as it accounts for:*   - *Intra-individual correlation of repeated measurements over time,* - *Inter-individual variability (random effects),* - *Missing data at some time points, which are common in clinical studies.*   *This flexible statistical approach is appropriate for examining longitudinal data, handling potentially unbalanced data with missing entries, and accounting for the repeated nature of measurements within each participant[6].*  *In the model, time (categorical: H0, H2, H4), group (intervention vs. control), and their interaction (group × time) will be included as fixed effects.*  *A random intercept will be included for each subject to model individual baseline differences.*  *The group × time interaction term will specifically test whether the change in PaCO₂ over time differs significantly between the two groups, providing a complementary view of the intervention’s effect beyond the primary endpoint.*   - ***Respiratory and cardiovascular parameters*** *(h0, h2, h4): Differences between groups over time will be analyzed using linear mixed models as for the evolution of PCO2.* - ***Respiratory comfort*** *(digital visual scale) and patient-reported outcomes (e.g., BDS, VNS) (h0, h2, h4): The evolution of these parameters from baseline (H0) to H4 will be conducted according to the same principles as for the analysis of the evolution of PCO2.* |
| .P12: Specify software used for sample size justification. | .The sample size was calculated using the BiostatTGV platform, which is based on the R statistical software environment. We have now clearly stated this in the revised manuscript. | ***.*The following sentence was added Line 413-415 :**  *“The sample size calculation was performed using Biostat TGV platform, an online tool that relies on the R statistical software for all underlying computations (https://biostatgv.sentiweb.fr/).”* |
| .Give reference for high dropout rate | .A 10% dropout rate was assumed based on previous clinical trials involving patients with AECOPD, particularly those evaluating NIV interventions. Similar studies have reported dropout or loss-to-follow-up rates in the range of 2–42% due to the clinical instability of this population and potential early discharge or withdrawal from interventions. We have now added a reference to support this estimate.  **Reference:** Park SY, Yoo KH, Park YB, Rhee CK, Park J, Park HY, Hwang YI, Park DA, Sim YS. The Long-term Efficacy of Domiciliary Noninvasive Positive-Pressure Ventilation in Chronic Obstructive Pulmonary Disease: A Meta-Analysis of Randomized Controlled Trials. Tuberc Respir Dis (Seoul). 2022 Jan;85(1):47-55. | **Reference added Line 409** |
| .Additional sample size increase for change in statistical test conflicts to power, validity and reproducibility of the trial. | . We agree that increasing the sample size solely to accommodate a potential change in statistical testing (e.g., from parametric to non-parametric methods) could raise concerns regarding the validity and reproducibility of the trial. We would like to clarify that the sample size adjustment in our protocol is not based on a change in statistical assumptions but is a conservative measure to account for possible participant drop-out. Our original power calculations and hypotheses remain unchanged. This approach aims to preserve the planned statistical power and robustness of our primary analysis despite potential attrition. We have revised the wording in the manuscript to better reflect this clarification and to avoid any misunderstanding regarding the rationale for the sample size increase. | **. Line 407-410**  *“Assuming an alpha level of 5% and a power of 80%, a minimum of 31 participants per group is required to detect a statistically significant difference in the reduction of PCO2 between the intervention and control groups. Accounting for a 10% dropout rate [43] the final sample size is set at 35 patients per group.”* |
| .P13: Change in statistical analysis procedure based on observed data conflicts to type one error rate.  Change from t-test to Mann-Whitney U test implies change in hypotheses.  The Shapiro Test has no power.  This approach does not maintain the type one error rate., i.e. conflicts to power, validity and reproducibility of the trial.  State the planned statistical model with parameters for primary endpoint variable analysis! | .We thank the reviewer for this important observation regarding the choice of statistical tests for the primary endpoint analysis. We fully acknowledge the risk of inflating the type I error rate when changing statistical tests based on observed data characteristics, and we have accordingly revised and clarified our statistical analysis plan to ensure methodological robustness and transparency   1- **Parametric approach justified by sample size:**   For the primary analysis of the change in PaCO₂ (ΔPaCO₂), we will use parametric methods, specifically an independent samples t-test and multivariable linear regression model to account for potential confounding variables. The justification for this choice is based on the **Central Limit Theorem**, which supports the assumption that the sampling distribution of the mean approximates normality when the sample size is sufficiently large. As our planned sample size exceeds 30 participants per group, parametric tests remain appropriate and statistically valid, even if the raw data are not perfectly normally distributed.   2- **Assessment of Distribution:**   To assess the distribution of continuous variables, we will not rely solely on the Shapiro-Wilk test, which, as the reviewer rightly notes, may lack power. Instead, we will complement it with **visual inspection methods** such as histograms and Q-Q plots to evaluate the normality assumption. This dual approach provides a more reliable and nuanced understanding of data distribution, supporting the appropriateness of parametric methods.   3- **Revised Statistical Analysis Plan:**   Considering the reviewer’s feedback, we have revised and clearly state the statistical analysis plan.  Sensitivity analyses will be performed to confirm the stability of findings. All analyses, including any sensitivity tests (e.g., non-parametric tests), will be clearly reported as secondary or exploratory and will not replace the pre-specified primary model. | **. The methods section was reformulated, and statistical analysis plan was added line 416-484.**  “**Statistical analysis**  All statistical analyses will be conducted in the intention-to-treat population at a two-sided 5% alpha risk.  For each group and at each assessment time, categorical variables will be summarized as numbers and percentages, and continuous variables will be expressed as number, mean, and standard deviation (SD). Quantitative variables with skewed distribution will be presented in terms of median and interquartile range (IQR) (25th-75th percentile). The normality of the distribution will be assessed using the Shapiro-Wilk test. In addition, histograms and Q–Q plots will be examined for each group to visually inspect the distribution.  **Primary Endpoint Analysis:**  The primary outcome is the change in PaCO₂, defined for each participant as the difference between baseline (t0) and the value measured immediately after the first NIV session (t4), calculated as: ΔPaCO₂ = PaCO₂ baseline – PaCO₂ at 4 hours.  To assess the impact of the music-based intervention, the mean ΔPaCO₂ will be compared between the intervention and control groups using an independent samples t-test, assuming normal distribution and homogeneity of variances. For the primary efficiency analysis, only sessions with the intended duration will be included.  **Adjusted Primary Analysis:**  To account for potential confounding variables, a multivariable linear regression model will be performed, with ΔPaCO₂ as the dependent variable and group allocation (intervention vs. control) as the main independent variable. The model will adjust for baseline factors known or suspected to influence PaCO₂ levels, including :  • Dyspnea severity (assessed by the mMRC scale),  • Prior use of home NIV,  • Obesity status,  • Kelly Mathey scale score, and  • Baseline anxiety trait (measured by the STAI trait score).  **Secondary Endpoints:**  - **Change of PaCO₂ over time:** To evaluate the temporal evolution of PaCO₂ (at H0, H2, and H4), a linear mixed-effects model (also referred to as multilevel models) will be employed. This statistical approach is particularly suited to repeated measures data, as it accounts for:  -Intra-individual correlation of repeated measurements over time,  -Inter-individual variability (random effects),  -Missing data at some time points, which are common in clinical studies.  This flexible statistical approach is appropriate for examining longitudinal data, handling potentially unbalanced data with missing entries, and accounting for the repeated nature of measurements within each participant [44].  In the model, time (categorical: H0, H2, H4), group (intervention vs. control), and their interaction (group × time) will be included as fixed effects.  A random intercept will be included for each subject to model individual baseline differences.  The group × time interaction term will specifically test whether the change in PaCO₂ over time differs significantly between the two groups, providing a complementary view of the intervention’s effect beyond the primary endpoint.  -Respiratory and cardiovascular parameters (h0, h2, h4): Differences between groups over time will be analyzed using linear mixed models as for the evolution of PCO2.  -Respiratory comfort (digital visual scale) and patient-reported outcomes (e.g., BDS, VNS) (h0, h2, h4): The evolution of these parameters from baseline (H0) to H4 will be conducted according to the same principles as for the analysis of the evolution of PCO2.  -Neurological and psychological assessments (scales) (h0, h4): Changes from baseline (H0) to the final time point (H4) will be analyzed within groups using paired t-tests. Between-group comparisons of these changes will be performed using independent t-tests.  -Unscheduled interventions: the number of unplanned interventions is a count outcome variable. The comparison groups will therefore be based on a Poisson regression model.  **Sensitivity analysis :**  To evaluate whether session duration affects the primary outcome a sensitivity analysis will be performed including all recorded sessions, regardless of length. A separate multivariable linear regression model will be used, with ΔPaCO₂ as the dependent variable, group allocation as the main predictor, and session duration included as an additional covariate. The model will also adjust for the same baseline confounders (mMRC scale, familiarity with home NIV, obesity status, Kelly-Mathey score, and STAI-Trait score). This analysis will help determine whether the duration of the session significantly modifies the effect of the intervention on PaCO₂ changes.  **Missing data:**  Missing data will be handled using multiple imputation techniques if the proportion of missing data is significant, ensuring unbiased estimates. Results will be interpreted with both statistical significance (p < 0.05) and clinical relevance considered.  The statistical analysis will be conducted using SPSS version 20. Analyses will be performed by an independent investigator (DC in the authors’ list) blinded to treatment allocation, ensuring unbiased interpretation of the data.” |
| .P14: Substitute bilateral by two-sided | .This has been corrected throughout the manuscript. | **Line 398 and line 417**  Done |
| .P14: methods against bias are not referred to | .We appreciate the reviewer’s emphasis on the importance of minimizing bias to ensure the internal validity of the trial. In response, we have clarified and reinforced throughout the revised manuscript the specific methodological measures implemented to reduce potential sources of bias. These include:   - **Centralized randomization with strict allocation concealment,** handled independently from recruitment and outcome assessment. - **Blinded outcome assessment**, performed by trained evaluators not involved in the intervention or recruitment - **Standardized data collection procedures**, using validated instruments at predefined time points. - **A pre-specified statistical analysis plan**, with clearly defined primary and sensitivity analyses to avoid data-driven decisions.   These revisions have been incorporated into the Methods section to enhance clarity and transparency. | ***.The following paragraphs were added***  ***L 168:*** *“The randomization sequence will be generated in advance by a research assistant independent of participant recruitment, clinical care, outcome assessment, or data.”*  ***L171****:” Concealment mechanism: To ensure strict allocation concealment and prevent allocation bias, the randomization sequence will be securely stored and inaccessible to all study investigators and site staff involved in screening, enrolment, or intervention delivery…..”*  ***L 179:*** *“This procedure ensures allocation concealment until the point of randomization, maintaining methodological integrity and minimizing allocation bias.”*  ***L186:*** *“blinding: Given the nature of the intervention, blinding of participants is not feasible, as they will know whether they are listening to music. Nevertheless, the study will follow the principles of the Prospective Randomized Open Blinded Endpoint (PROBE) design [10], which is specifically intended to minimize bias in outcome assessment and data analysis.*  *To maintain blinding integrity and minimize bias, only the participant and the designated research assistant responsible for* *opening the allocation envelope and administering the assigned intervention will be aware of the group assignment.”*  ***L308****: “To minimize bias, these residents will not be involved in recruitment or intervention delivery. All assessors will undergo specific training to standardize data collection procedures and reduce inter-rater variability.”*  ***L 478:*** *“Missing data:*  *Missing data will be handled using multiple imputation techniques if the proportion of missing data is significant, ensuring unbiased estimates. Results will be interpreted with both statistical significance (p < 0.05) and clinical relevance considered.*  *The statistical analysis will be conducted using SPSS version 20. Analyses will be performed by an independent investigator (DC in the authors’ list) blinded to treatment allocation, ensuring unbiased interpretation of the data”* |

**References :**

1.	Rochwerg B, Brochard L, Elliott MW, Hess D, Hill NS, Nava S, et al. Official ERS/ATS clinical practice guidelines: noninvasive ventilation for acute respiratory failure. Eur Respir J. 2017;50: 1602426. doi:10.1183/13993003.02426-2016

2.	Chawla R, Dixit SB, Zirpe KG, Chaudhry D, Khilnani GC, Mehta Y, et al. ISCCM Guidelines for the Use of Non-invasive Ventilation in Acute Respiratory Failure in Adult ICUs. Indian J Crit Care Med Peer-Rev Off Publ Indian Soc Crit Care Med. 2020;24: S61. doi:10.5005/jp-journals-10071-G23186

3.	Wang CC, Emrich M, Rives H, Ovalles A, Wright D, Wyka K, et al. Music interventions for posttraumatic stress disorder: A systematic review. J Mood Anxiety Disord. 2024;6: 100053. doi:10.1016/j.xjmad.2024.100053

4.	Messika J, Martin Y, Maquigneau N, Puechberty C, Henry-Lagarrigue M, Stoclin A, et al. A musical intervention for respiratory comfort during noninvasive ventilation in the ICU. Eur Respir J. 2019;53. doi:10.1183/13993003.01873-2018

5.	Duca A, Rosti V, Brambilla AM, Cosentini R. Non-invasive ventilation in COPD exacerbation: how and why. Intern Emerg Med. 2019;14: 139–142. doi:10.1007/s11739-018-1974-z
